# Effects of Process and Formulation Parameters on Submicron Polymeric Particles Produced by a Rapid Emulsion-Diffusion Method

**DOI:** 10.3390/nano12020229

**Published:** 2022-01-11

**Authors:** Clara Luisa Domínguez-Delgado, Zubia Akhtar, Godfrey Awuah-Mensah, Braden Wu, Hugh David Charles Smyth

**Affiliations:** Division of Molecular Pharmaceutics and Drug Delivery, College of Pharmacy, The University of Texas at Austin, 2409 University Avenue, Austin, TX 78712, USA; zubiaakhtar@utexas.edu (Z.A.); gamensah@utexas.edu (G.A.-M.); braden.s.wu@gmail.com (B.W.)

**Keywords:** pH-sensitive nanoparticles, rapid emulsification-diffusion, solubility parameter correlation, Eudragit^®^ E100, PLGA, biodegradable nanoparticles

## Abstract

Emulsification-diffusion method is often used to produce polymeric nanoparticles. However, their numerous and/or lengthy steps make it difficult to use widely. Thus, a modified method using solvent blends (miscible/partially miscible in water, 25–100%) as the organic phases to overcome these disadvantages and its design space were investigated. To further simplify the process, no organic/aqueous phase saturation and no water addition after the emulsification step were performed. Biodegradable (PLGA) or pH-sensitive (Eudragit^®^ E100) nanoparticles were robustly produced using low/medium shear stirring adding dropwise the organic phase into the aqueous phase or vice versa. Several behaviors were also obtained: lowering the partially water-miscible solvent ratio relative to the organic phase or the poloxamer-407 concentration; or increasing the organic phase polarity or the polyvinyl alcohol concentration produced smaller particle sizes/polydispersity. Nanoparticle zeta potential increased as the water-miscible solvent ratio increased. Poloxamer-407 showed better performance to decrease the particle size (~50 nm) at low concentrations (≤1%, *w*/*v*) compared with polyvinyl alcohol at 1–5% (*w*/*v*), but higher concentrations produced bigger particles/polydispersity (≥600 nm). Most important, an inverse linear correlation to predict the particle size by determining the solubility parameter was found. A rapid method to broadly prepare nanoparticles using straightforward equipment is provided.

## 1. Introduction

Emulsification-solvent diffusion method is able to produce polymeric nanoparticles (NPs) with high yields, no need of high shear stirring or ultrasonication, batch-to-batch reproducibility and good encapsulation efficiencies [1,2]. However, numerous and long steps involved to pre-saturate the solvents and to eliminate higher volumes of water from the final dispersion make it difficult to use as a feasible method for many applications. Moreover, the tunability or range of particles sizes produced from the few partially miscible solvents is limited [3]. Thus, modifications of this method are proposed in an attempt to overcome those disadvantages. Additionally, the seeking of simple, eco-friendly, safe and scalable methods to produce NPs are more often required in the pharmaceutical field.

Biomedical applications of polymeric NPs have been growing, especially in recent years due to their multifunctional properties [4]. Generally, methods to produce NPs involve two main strategies, the use of preformed polymers or the polymerization process using monomers [5]. Preformed polymers are preferred because of the low toxicity compared with the polymerization techniques [3,6]. The “top-down” method for producing polymeric nanoparticles includes solvent emulsification–evaporation, solvent emulsification–diffusion, coacervation, and nanoprecipitation (solvent displacement method) [2,5].

In the standard emulsification-diffusion method, an o/w emulsion is formed using a partially water-miscible solvent previously saturated with water at room temperature to ensure the initial thermodynamic equilibrium of both liquids. This organic phase (OP), containing the polymer and the drug, is emulsified with an aqueous phase (AP) previously saturated with the organic solvent, containing a surfactant. The subsequent dilution with water induces the solvent diffusion from the droplets to the external phase, resulting in the formation of NPs. The solvent is then removed by evaporation or filtration [1]. This method was based on the emulsification-evaporation method in which non water-miscible solvents (generally, dichloromethane and chloroform) are used to prepare an emulsion with the subsequent solvent removal [7]. However, in order to use less toxic solvents and low shear stirring, the emulsification-diffusion method was further developed. Several modifications of this method have been achieved (Table 1) skipping or adding some steps [8], changing operating conditions in some steps [9], using different organic solvents [10,11,12], and replacing mixing techniques [13,14,15]. Similar modifications to the emulsification-solvent evaporation method have also been reported [16,17,18]. All these modifications have been performed in an attempt to provide new and better alternatives to produce NPs. Nevertheless, the use of toxic solvents (Class I) [19] and several as long steps are still involved in the processes.

In this study, the emulsion-diffusion method was modified to produce pH-sensitive polymeric or biodegradable NPs by using solvent blends as organic phases (OPs), consisting of partially water-miscible solvents and water-miscible solvents at ratios from 25 to 100%. Solvents Class 3 (low toxicity) [19] were used in this study. No previous solvents saturation and no water addition after the emulsification step were done, reducing the steps and consequently the time of NPs processing using simple equipment. Two polymers (Eudragit^®^ E100 and PLGA 85:15 and 50:50) and two stabilizers, poloxamer-407 (P-407) and polyvinyl alcohol (PVA), were chosen for their safety [20,21] and used varying their concentrations. NPs average size, polydispersity index (PdI), zeta potential and pH analysis were determined to evaluate the influence of the formulation and operating parameters. Surface-response plots to analyze the design space of the method were built. More importantly, a linear correlation to predict the particle size in the final aqueous phase from the Hansen solubility parameters of the organic solvent blends was investigated. These studies were performed since the particle dispersibility [22,23], transmittance [24] or particle sizes have only been studied in organic solvents [25] from the Hansen solubility parameters.

## 2. Materials and Methods

### 2.1. Materials

Poly (DL-lactide-co-glycolide) 85:15 (Mw 50,000–75,000, viscosity range: 0.55–0.75 dL/g in CHCl3) and 50:50 (viscosity range: 0.55–0.75 dL/g in HFIP, Mw 30,000–60,000) ester terminated (PLGA); and dimethylaminoethyl methacrylate (Eudragit^®^ E 100) were provided by Lactel, Durect Corporation (Birmingham, AL, USA) and Evonik Röhm GmbH (Darmstadt, Germany), respectively. They were used as the main polymers to constitute the nanoparticles. Polyvinyl alcohol 4–88, (PVA, EMPROVE^®^), Poloxamer 407 (P-407, Lutrol^®^ F127 NF M) were purchased from Merck KGaA, EMD Millipore Corporation (Waltham, MA, USA); BASF SE, Ludwigshafen, Germany); and BASF Corporation (Florham Park, NJ, USA), respectively, and they were used as stabilizers. Solvent mixtures of the following compounds HPLC grade were used as OP: Polyethylene glycol 400 (PEG 400 MW), obtained from Spectrum Chemical MFG CORP (New Brunswick, NJ, USA); Propylene glycol and benzyl alcohol (BnAl), acquired from Sigma-Aldrich Chemie GmbH, (Steinheim, Germany); Acetone (AC), methyl ethyl ketone (MEK), methylene chloride (MC), ethanol (Et), tetrahydrofuran (THF) and ethyl acetate (EtAc), purchased from Thermo Fisher Scientific (Branchburg, NJ, USA). Distilled and deionized water were used.

### 2.2. Experimental Methods

#### 2.2.1. Pre-Optimization of the Method to Produce Nanoparticles

Biodegradable particles of PLGA (85:15 or 50:50) and pH-sensitive nanoparticles of Eudragit^®^ E100 were prepared by a modification of emulsion-diffusion process. These polymers and the stabilizers poloxamer 407 (P-407) and polyvinyl alcohol (PVA) were chosen for their safety [20,21] and their wide use in nanoparticles. Firstly, and in order to skip the solvent saturation step, previous solubility and batches tests were carried out in different solvents and concentrations or using their blends (Table 2). In these first tests, the polymer PLGA (85:15) and (50:50) at <2% (*w*/*v*) showed poor solubility with PEG 400 MW and Et as OP. Probe ultrasonication, able to improve their solubility in other study [26], was not used in order to develop a simple method.

On the other hand, THF with Et and high volumes of MC as OPs, produced coarse aggregates (Figure S1 of the Supplementary Material). Thus, MC was not further used because of its considerable toxicity [27]. Probes with solvent blends of partially water miscible solvents and water miscible solvents, as OPs, were able to dissolve PLGA (50:50 or 85:15). Here, attempts to produce nanoparticles varying the stirring type were performed. Magnetic stirring (10 min) used to emulsify the OPs and APs, skipping the water addition after the emulsification process and followed by the organic solvent removal by vacuum steam distillation at 30 °C, produced heterogeneous microparticles with an unwanted film. A little improvement was obtained using PLGA 50:50 instead PLGA 85:15 (due to its higher polymer hydrophilicity and solubility [28]) or using THF (100%) or decreasing the polymer concentration (Table 2).

With the intention to obtain unimodal particle size distributions in the nanometer scale, magnetic stirring (10 min) followed by high shear stirring (IKA Ultra-Turrax^®^ T 25, IKA Works, Inc., Wilmington, NC, USA) at 8000 rpm/10 min were probed. However, after the solvent removal step, two particle size distributions around 200 nm and 1200 nm were found (batch ***g***, Table 2). Finally, an unimodal particle size distribution around ~190 nm was achieved only adding dropwise (2 mL/min) the THF-EtAc (50:50) blend as OP into the AP simultaneously stirred with mechanical high shear stirring at 8000 rpm for 10 min (batch ***j***, Table 2). All other operating conditions were kept constant (detailed results available in Figure S1 of the Supplementary Material).

#### 2.2.2. Nanoparticle Preparation and Experimental Design

Once the method to prepare nanoparticles was pre-optimized, 12 experimental designs (Table 3) were performed in order to determine the method space design and the influence of the formulation and operating parameters. All formulations from these experimental designs were prepared by dissolving the polymer (PLGA (50:50) or Eudragit^®^ E100 in the OPs, consisting of solvent blends of partially water-miscible solvents and water-miscible solvents. THF-EtAc, ACE-MEK, ACE- EtAc and Et-EtAc were used as solvent blends. These OP solutions were added dropwise (2 mL/min) into an aqueous solution containing PVA or P-407 constantly stirred, used as stabilizers. The OP and AP volumes were kept constant in all batches with a ratio of 1:2, respectively. The emulsification was performed using either a mechanical stirrer Caframo Model BDC3030 (Georgian Bluffs, ON, Canada) with a 4-bladed propeller stirrer or under mechanical high shear stirring (IKA Ultra-Turrax^®^ T 25, IKA Works, Inc., Wilmington, NC, USA) at 8000 rpm for 10 min. No water addition was done after the emulsification step. Finally, the organic solvent was removed by vacuum steam distillation at 30 °C.

Thus, the effect of using solvent blends miscible and partially miscible in water, with different polarity/acidity, varying their ratios, and using high shear stirring in the emulsification step, on size, PdI and zeta potential of PLGA nanoparticles was studied (Experimental design 1, Table 3).

Regarding Eudragit^®^ E100, it was used to prepare nanoparticles in the experimental designs 2–6 and 8. The effect of increasing the polymer concentration varying the water-miscible solvent ratio in the solvent blend (Experimental design 2, Table 3), and the effect of using a different water-miscible solvent varying its ratio in the solvent blend, on the size, PdI and zeta potential of Eudragit^®^ E100 nanoparticles were investigated (Experimental design 3, Table 3). A three factor (two stabilizers, three stabilizer concentrations and four ratios of the solvent blend as OP) experimental design was also carried out to evaluate the behavior on the particle sizes, PdIs, electrical charges and the pH (Experimental design 4, Table 3). Batches with increasing stirring rates in a feasible range to emulsify the AP and OP were manufactured in order to determine the range to produce particles in the nanometer scale by using a mechanical stirrer Caframo Model BDC3030 (Georgian Bluffs, ON, Canada) with a 4-bladed propeller stirrer (Experimental design 5, Table 3). The effect of the use of a higher polymer concentration, versus the use of a more polar solvent blend, varying its ratios into the OP, (Experimental design 6, Table 3) was studied. The effect of varying the concentration of polymer and stabilizer (Experimental design 8, Table 3) was also evaluated.

PLGA (50:50) was also used in the experimental designs 7 and 9–12, (Table 3). The influence in the order of the aqueous and organic phase’s addition in the emulsification step on the particle size, PdI and Zeta potential was studied (Experimental design 7). The effect of the three lowest stabilizer concentrations (Experimental design 9) and the highest stabilizer concentration versus two solvent blend ratios (Experimental design 10) were also studied. Finally, the effect of using two solvents blends with different polarity varying their ratios (Experimental design 11), as well as the effect of using two different stabilizers (Experimental design 12) on the particle size distribution, electrical charges and pH were analyzed. Surface-response plots from all experimental designs were built to analyze the design space of the method. All the operating and formulation parameters used in the experimental designs are shown in Table 3.

#### 2.2.3. Particle Size, Charge and pH Determination

NPs average size, polydispersity index and zeta potential analysis of the NPs were determined using a Zetasizer (Nano series Nano-ZS, Malvern Panalytical, Enigma Business Park, Malvern, UK). Measurements for all batches were conducted at 25 °C, at the water viscosity and using dynamic light scattering with non-invasive backscatter optics (NIBS, *n* = 3). Zeta potential of all batches was determined at 25 °C using the viscosity and dielectric constant of water, using deionized water as a dispersion medium.

Dispersions were diluted with distilled water to ensure that the count rate of particles was within the sensitivity range of the instrument for all measurements (*n* = 3). NP final suspensions prepared with Eudragit^®^ E100 and solvent blends of Et-EtAc (0:100) at ratios from 25 to 100% were used to determine the pH, as well as the suspensions obtained in the experimental designs 11 and 12 (Table 3) using PLGA (50:50). Measurements were directly collected from the NP suspensions at constant and moderate stirring after suitable pH-meter calibration (*n* = 3).

#### 2.2.4. Determination of Solubility Parameters of the Nanoparticles Components

Solubility parameters of the solvent blends were calculated and used for comparison to study the correlation with the nanoparticle formation in this study. The solubility parameter was introduced by Hildebrand and Scott to determine an estimation of the drug/excipient miscibility [29]. Hildebrand defined the solubility parameter, *δ_T_*, as square root of the cohesive energy density, *E_P_*, of a substance. This is the energy required to separate or attract the atoms or molecules from each other.

(1) $$\Delta \delta_{T}={\left(\Delta H_{v}-RT/V_{m}\right)}^{0.5}$$

where ∆*H_v_* is the heat of vaporization, *R* is the gas constant, *T* is the temperature and the molar volume is *V_m_*. This equation was developed for simple liquid mixtures and their use is limited for non-polar solvents. Derivatives approximations of Hildebrand’s equation have been described to better establish the interactions between substances. One of these is the Hansen approach [30]. *H_aSP_* is derived from three different solvent energies: *E_D_* measured as the intermolecular dispersion energy, *E_P_* as the dipolar intermolecular energy, and *E_H_* as the hydrogen bonding energy. The sum of the square energies divided by the molar volume, *V_m_*, is the square of the total solubility parameter [30],

(2) $$\delta_{T}{}^{2}=\delta^{2}{}_{HaSP}=\delta_{D}{}^{2}+\delta_{P}{}^{2}+\delta_{H}{}^{2}$$

*δ*^2^*_HaSP_* of the pure solvents were obtained from the literature [31] and the solubility parameters of the solvent blends were calculated as the sum of the solubility parameter values of the pure solvents by their volume fraction as previously reported [32]. The calculated solubility parameters of the solvent blends were plotted with the particle size values from batches prepared with the respective solvent blends used as OPs at the same operating condition. A linear regression analysis was performed, and the correlation coefficient was determined.

#### 2.2.5. Statistical Analysis

The results were statistically evaluated by analysis of variance (ANOVA) one-way to compare two or multiple groups. Post-hoc comparisons of individual group means of one or more factors were performed applying the Bonferroni test. Differences were considered significant if *p* value < 0.05. When variances of multifactorial groups were different, analysis per individual factor was performed. Models were constructed to produce three-dimensional response surface plots to analyze the influence of each response using data from the experimental designs with different factors and levels (Table 3). Software STATGRAPHICS Centurion XVI was used for this purpose.

## 3. Results and Discussion

A rapid emulsion-diffusion method to prepare polymeric nanoparticles (pH-sensitive polymeric or biodegradable) for general applications was achieved. This reduced method was performed by using aqueous and organic phases with no previous mutual saturation. The OPs consisted of partially water-miscible solvents and water-miscible solvents varying their ratios in order to produce nanoparticles of 50 ≤ 1000 nm, using either low or high shear stirring (1500–8000 rpm) from straightforward equipment. No water addition after the emulsification step was carried out, reducing the time of NPs processing (Table 1, Table 2 and Table 3; detailed results can be found in Table S1 of the supplementary material). An important linear correlation was found to predict the particle size from the solubility parameters of the OPs (Table 4).

### 3.1. Pre-Optimization of the Rapid Emulsion-Diffusion Method

A single mode distribution of the batch (particles below 200 nm and PdI of 0.054) was only achieved by adding dropwise the OP into the AP or vice versa using high shear stirring at 8000 rpm, as shown in Table 2. This contrasts with the results from a previous study using the standard method in which the dropwise addition or total addition of the OP into the AP did not influence the particle size distribution [2]. Systems magnetically stirred for 10 min adding dropwise the OP into the AP and subsequently stirred using a homogenizer of high shear at 8000 rpm for 10 min, were not able to produce unimodal particle size distributions. Two populations in the micro and nanoscale were obtained.

### 3.2. Influence of the Process and Formulation Parameters

Interesting results are found from the experimental designs determining the space design in which NPs can be formed using this rapid emulsion-diffusion method. Biodegradable or pH-sensitive nanoparticles (<1000 nm) were robustly produced by using solvent blends consisting of partially water-miscible solvent and water-miscible solvents, as OPs, to be emulsified with an AP containing a stabilizer. Low shear stirring (1500–3000 rpm) with a mechanical stirrer or high shear stirring (8000 rpm) with an Ultra Turrax^®^ stirrer were good enough to produce nanoparticles. The dropwise addition of the OP into the AP or the inverse order in the emulsification step was able to produce nanoparticles, though smaller particle sizes were obtained in the first case. Important tendencies and a linear correlation to decrease the particle size by increasing the solubility parameter were found. This suggests that the formation of the nanoparticles is given in the emulsification step and a prediction of the particle size can be done by knowing the OP solubility parameter.

**Figure 1:**
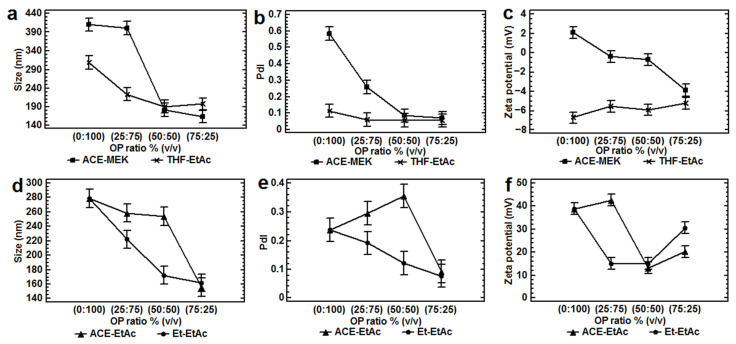
Influence of different organic phases at different ratios on the particle size, polydispersity index and zeta potential by using two polymers: (**a**–**c**) PLGA (50:50), 8000 rpm; (**d**–**f**) Eudragit^®^ E100, 2000 rpm. All batches were prepared with 200 mg of polymer, a ratio of OP:AP (1:2), PVA 2%, *n* = 3. All the means bars correspond to the Bonferroni interval at the 95.0% confidence level.

**Figure 2:**
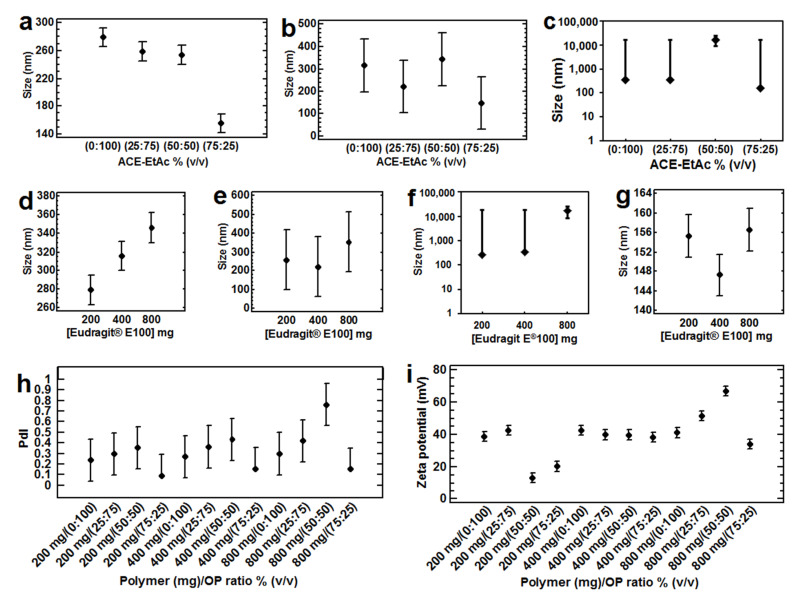
Influence of the polymer concentration (**a**) 200 mg; (**b**) 400 mg and (**c**) 800 mg and the OP ratio of ACE-EtAc (**d**) 0:100; (**e**) 25:75; (**f**) 50:50 and (**g**) 75:25% (*v*/*v*) on the particle size (**h**) PdI and (**i**) zeta potential. All batches were prepared with Eudragit^®^ E 100, ACE-EtAC as OP, a ratio OP:AP (1:2), 2000 rpm, PVA 2%, *n* = 3 (Experimental design 2). All the means bars correspond to the Bonferroni interval at the 95.0% confidence level.

**Figure 3:**
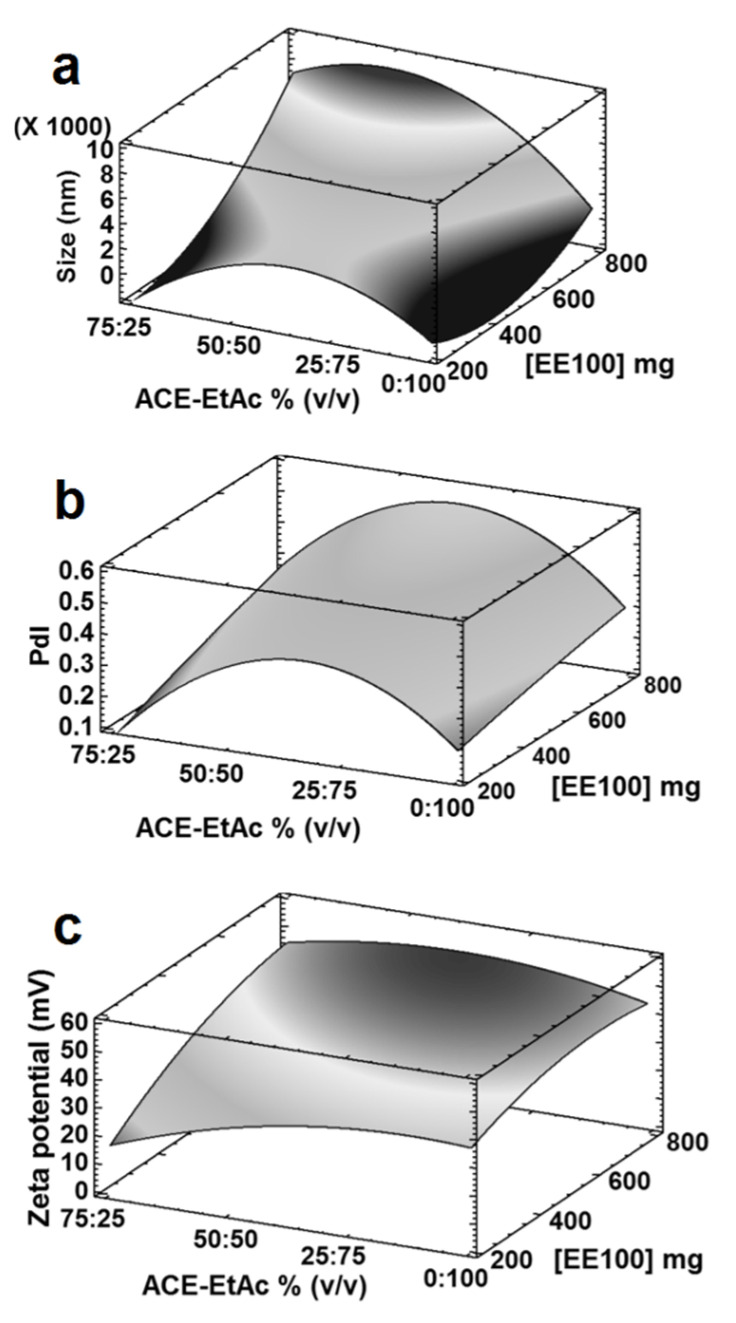
Three-dimensional model showing the response-surface estimated; (**a**) particle size, (**b**) PdI and (**c**) zeta potential by using ACE-EtAc, as OP, varying its ratios, and Eudragit^®^ E 100 (EE100) at three concentrations (Table 3, design 2). All batches were prepared with a ratio OP:AP (1:2), 2000 rpm, PVA 2%, *n* = 3.

**Figure 4:**
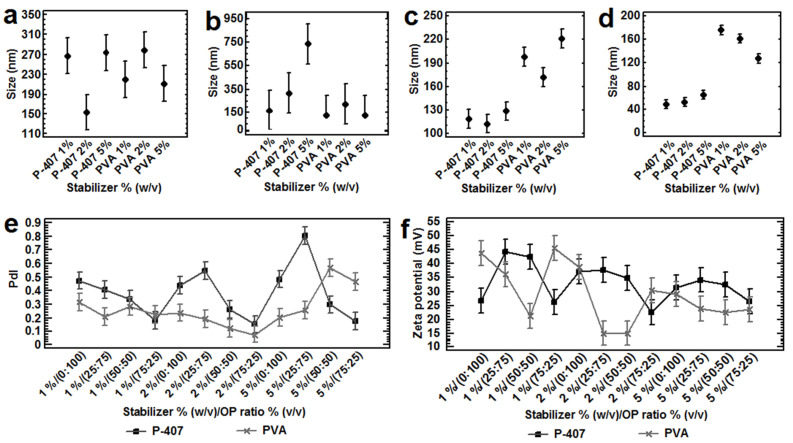
Influence of the OP ratio of Et-EtAC at (**a**) 0:100; (**b**) 25:75; (**c**) 50:50 and (**d**) 75:25, type of stabilizer and its concentration % (*w*/*v*) on the (**a**–**d**) particle size, (**e**) PdI and (**f**) zeta potential. All batches were prepared with 200 mg of Eudragit^®^ E 100, a ratio OP:AP (1:2), 2000 rpm, *n* = 3 (experimental design 4). All the means bars correspond to the Bonferroni interval at the 95.0% confidence level.

**Figure 5:**
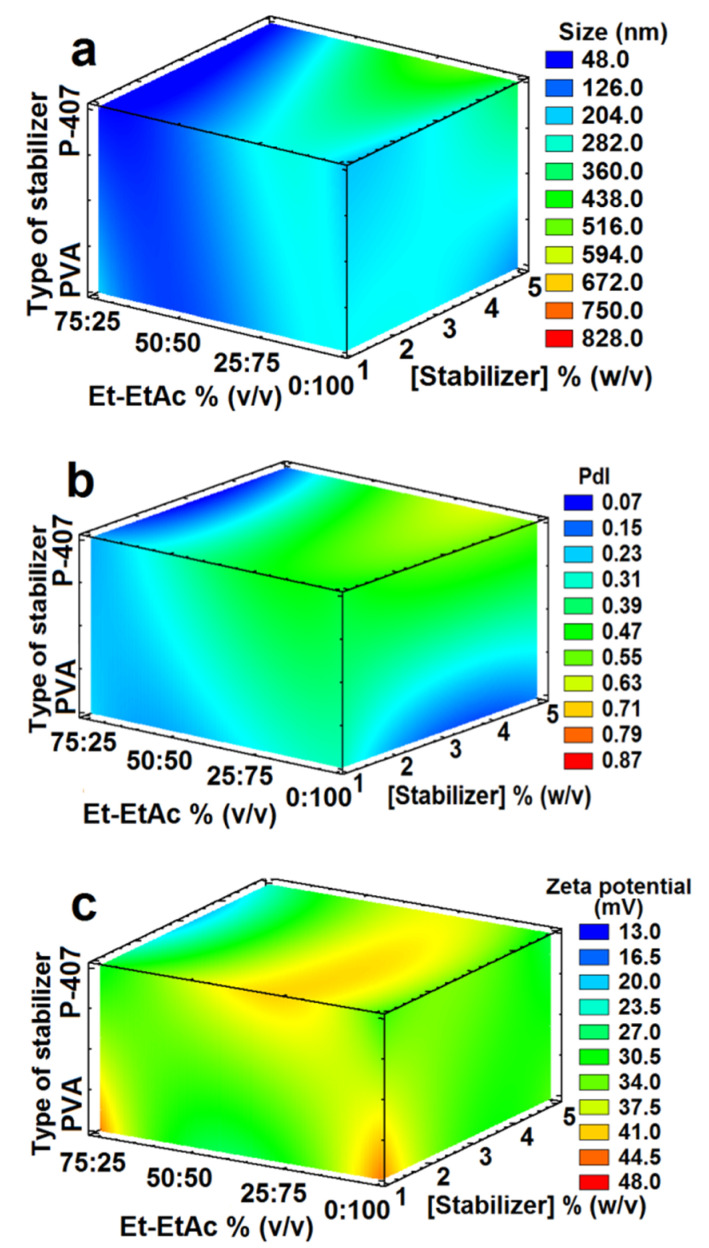
Three-dimensional model showing the response-surface estimated; (**a**) particle size, (**b**) PdI and (**c**) zeta potential by using Et-EtAc varying its ratios and two stabilizers (P-407 or PVA) also varying their concentrations (Table 3, Experimental design 4). All batches were prepared with 200 mg of Eudragit^®^ E 100, a ratio OP:AP (1:2), 2000 rpm, *n* = 3, *p* < 0.05.

**Figure 6:**
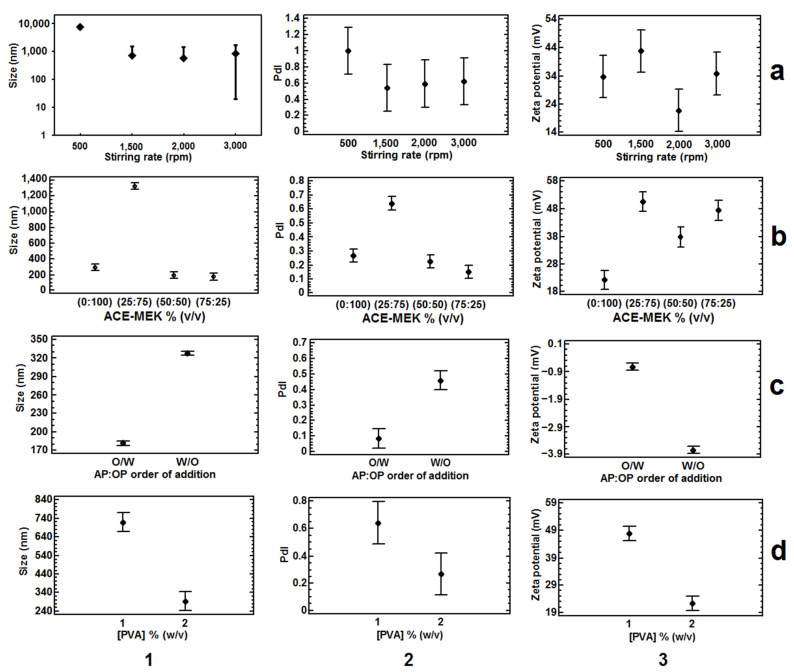
Influence of (**a**) the stirring rate (Experimental design 5); (**b**) the solvent blend ratios using 400 mg of Eudragit^®^ E 100 (Experimental design 6); (**c**) the AP:OP order of addition (Experimental design 7); and (**d**) the stabilizer concentration using 400 mg of Eudragit^®^ E 100 (Experimental design 8) on the (**1**) particle size, (**2**) PdI and (**3**) Zeta potential. All batches were prepared with a ratio OP:AP (1:2). All the bars of all means correspond to the Bonferroni interval at the 95.0% confidence level, *n* = 3.

**Figure 7:**
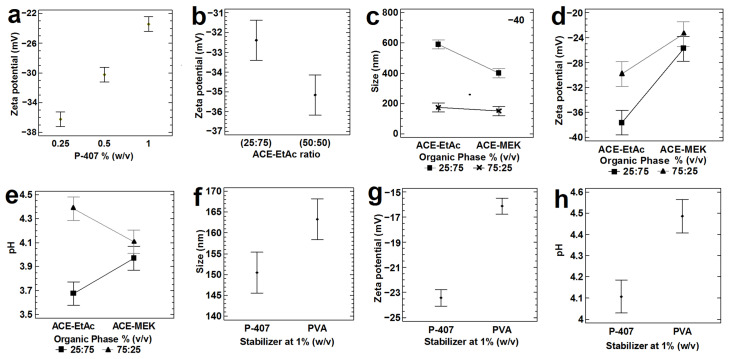
Influence of (**a**) P-407 at three low concentrations using ACE-MEK (75:25), Experimental design 9; and (**b**) ACE-EtAc at two ratios using P-407 5% (*w*/*v*; Experimental design 10), on the zeta potential. Effect of two solvent blends, as OP, at two ratios on (**c**) the particle size, (**d**) zeta potential and (**e**) pH by using P-407 1%, as stabilizer (Experimental design 11). Influence of P-407 and PVA at 1%, on the (**f**) particle size, (**g**) zeta potential and (**h**) pH, by using the solvent blend ACE-MEK (75:25), Experimental design 12. All batches were prepared with 200 mg of PLGA (50:50), 2000 rpm, and a ratio of OP:AP (1:2), *n* = 3. All the means bars correspond to the Bonferroni interval at the 95.0% confidence level.

Among the trends observed, the decrease in the partially water-miscible solvent ratio and simultaneously increasing the water-miscible solvent, increasing the OP polarity, or using higher concentrations of PVA or lower concentrations of P-407 with higher ratios of the water-miscible solvent, produced smaller sizes and PdIs using either Eudragit^®^ E 100 or PLGA (50:50). In contrast, smaller sizes and PdIs were obtained using 100% (*v*/*v*) of a less polar partially water-miscible solvent (i.e., EtAc compared with MEK). Using either Eudragit^®^ E 100 or PLGA 50:50, a wide range of particle sizes could be obtained (~50 ≥ 600 nm) when P-407 was used. At its lowest concentration however (1%, *w/v* P-407), smaller particle sizes were obtained compared with polyvinyl alcohol across the tested concentration range (1–5%, *w*/*v*; Figure 1, Figure 2, Figure 3, Figure 4, Figure 5, Figure 6 and Figure 7). In general, similar particle sizes were found using the closest solubility parameters of the solvent blends/polymers/stabilizers. Using higher ratios of a water-miscible solvent into the OP or increasing the stabilizer concentration, lower absolute values of NPs zeta potential were obtained with either Eudragit^®^ E 100 or PLGA (Table 3). A higher ionization of the polymeric molecules was confirmed measuring the pH from these nanoparticle aqueous dispersions. All the explanations to these effects on the responses studied are provided in detail in the following subsections.

#### 3.2.1. Effect of the Solvent Blend (OP) Composition/Ratio on Particle Size and PdI

In general terms, smaller sizes and PdIs were found increasing the solubility of either polymer (PLGA or Eudragit^®^ E 100) in the organic phase. The solubility as a factor to modulate the particle size was analyzed using the relative polarity, tension surface and the solubility parameter, from the existing literature, of the components used to produce the nanoparticles in this study. The relative polarity used [33] is the empirical normalized solvent parameter E^N^_T_, derived from the solvatochromism or transition energy at 25 °C of the long-wavelength visible absorption of a standard of a pyridinium N-phenolate betaine dye, which decreases as the polarity decreases [34]. Regarding the tension surface, a correlation has also been reported between the increase in the surface tension of organic solvent to produce a smaller contact angle and induce an easier imbibition of water due to the decrease in the interfacial tension between water and organic solvent [35]. On the other hand, according to Hildebrand’s solubility parameter approach, a higher mutual solubility will be given if the solubility parameter values of the solvents/polymers are closer [29].

Analyzing the systems with PLGA (50:50) and PVA at 2% (*w*/*v*), in which the partially water-miscible solvent was used at 100% (*w*/*v*), interesting results were obtained. Significantly (*p* < 0.05) smaller nanoparticles (~300 nm) and PdIs (~0.1) were found using EtAc (relative polarity = 0.228 [33,34] and surface tension = 23.2 mN/m at 25 °C [36]), a less polar partially water-miscible solvent, compared with MEK (relative polarity = 0.327 [33,34] and surface tension = 24.0 mN/m at 25 °C [36]) as OP. This effect was also evident at higher ratios (75%, *v*/*v*) of this less polar partially water-miscible solvent (EtAc) mixed with 25% (*w*/*v*) of THF into the OP (Table 3, Experimental design 1; Figure 1a,b). Similar results were found in other studies using PLGA (50:50) and ethyl acetate (relative polarity: 0.228 [33,34]) compared with methylene chloride (relative polarity: 0.309 [33,34]) by emulsification-evaporation [16] and emulsion–diffusion-evaporation methods [11]. The same behavior was found using Eudragit^®^ E100, PVA at 2% (*w*/*v*) and the same partially water-miscible solvents at 100% (*w*/*v*; Table 3, Experimental design 2 and 5; Figure 1d and Figure 6a). The higher particle sizes obtained with MEK as OP could be due to its higher surface tension compared with that of the EtAc.

In contrast, using P-407 at 1% (*w*/*v*), PLGA 50:50 and a more polar solvent blend as OP, consisting of 25:75 of ACE-MEK compared with ACE-EtAc (ACE relative polarity = 0.355; surface tension = 23.0 mN/m at 25 °C [36]), significantly smaller sizes (around 400 nm, *p* < 0.05) and PdIs (0.5) were found. Moreover, in PLGA systems stabilized with either PVA or P-407, a decrease in the particle size below 200 nm and PdI around 0.1 was also found decreasing the ratio of the partially water-miscible solvents into the OPs and simultaneously increasing the ratios of the water-miscible solvents ACE or THF (THF relative polarity = 0.207 [33,34], and surface tension: THF = 26.7 mN/m at 25 °C [36]; ACE relative polarity 0.355 [33,34]; and surface tension = 23.0 mN/m at 25 °C [36]) in the OPs (Table 3, Experimental design 1, Figure 1a,b,d,e; Experimental design 11, Figure 7c). More results can be found in the Figure S2 of the Supplementary Material.

When Eudragit^®^ E100 was used, similar trends were found. By using a more polar water-miscible solvent (Ethanol: relative polarity = 0.654 [33,34]) in the solvent blend Et-EtAc, compared with ACE-EtAc (ACE relative polarity 0.355 [33,34]), statistically significant (*p* < 0.05) smaller sizes (around 200 nm) and PdIs (0.2) were found (Table 3, Experimental design 3; Figure 1d,e). The same tendency to decrease the particle size was also found, by decreasing the ratio of the partially water-miscible solvents and increasing simultaneously the water-miscible solvents in the OPs and using either PVA or P-407 (Table 3, Experimental designs 2, 3 and 4; Figure 1d,e, Figure 2a,c,h, Figure 3a,b, Figure 4a–e and Figure 5a,b). This effect was more evident using 200 mg of Eudragit^®^ E100 instead of 400 mg (Table 3, Experimental design 2 and 6; Figure 2a–c, Figure 3a and Figure 6b). This agrees with the results found in another study using the blend chloroform:ethanol as OP and the emulsification-diffusion process [18]. Nevertheless, NPs could not be produced in other study with ACE (saturated with glucose)-EtAc at ratios of 70:30 and 80:20 by emulsion–diffusion–evaporation method [11].

These trends were evaluated plotting the solubility parameter calculated for each ratio of each solvent blend, used as OP, versus the particle size values (Table 4 and Table 5). The results agree with the above explanation, in which the decrease in the partially water-miscible solvents into the OPs, or the increase in the solubility parameter of the solvent blend produces the particle size decrease. The linear correlation could be only observed in batches prepared with the lowest polymer concentration (200 mg) either with PLGA 50:50 or Eudragit^®^ E 100 and using ≤2% (*w*/*v*) of either PVA or P-407. For these system compositions, the prediction of the particle size can be achieved knowing the solubility parameter of the OP. This correlation between the solubility parameters of the solvent blends and the particle size in aqueous dispersion in the nanoscale inversely correlates with those results obtained by the other group [25] for microparticles in organic solvents as dispersion medium, using monomers and the dispersion polymerization method. Those results showed that bigger particles were obtained as the solubility parameter increased [25]. Thus, with the correlation found in this study, it could be hypothesized that the formation of the nanoparticles is given in the emulsification step, in which all the components are interacting. The water addition after the emulsification [1] or the solvent removal [8] steps were stated for particle formation by other methods.

From all the experimental designs, some results in common were found. Using the ratios of 75:25 of the solvent blends THF-EtAc, ACE-EtAc and ACE-MEK with PLGA or Et-EtAc and ACE-EtAc with Eudragit^®^ E100, similar and smallest particle sizes and PdIs could be obtained in this study. This could be due to the similarity of the solubility parameters of the solvents blends with the polymers (solubility parameter of Eudragit^®^ E: 19.70 [37], and PLGA: 20.20 [32]) and the stabilizers (solubility parameter of PVA: 23.72 and P-407: 21.75 [37]). In contrast, using the solvent blend ACE-EtAc (50:50) as OP, and using either PLGA with P-407 at 5% (*w*/*v*) or Eudragit^®^ E100 at 800 mg, the most unstable and polydispersed systems were produced with particle sizes in the microscale (Table 3, Experimental design 2, Figure 2c,f; Experimental design 10, Figure S2c,d of the Supplementary Material). In the same way, the lowest linear correlations were found with these systems. It could be explained because the high polymer concentration (polymers/stabilizers) in those systems produced a decrease in the polymer-solvent interactions, increasing the polymer-polymer attraction forces, generating zones of polymeric chains in coiled conformation, unable to form monodispersions. Since the molecular mobility was restricted, the intermolecular friction increased, which could produce bigger particles. It is important to note that further studies must be done to analyze the correlation of the complete contribution of the solubility parameters of all components versus the particle size values to confirm the results obtained in this investigation.

Regarding the yield, it was apparently affected by the high vapor pressure and low boiling point of the solvents, particularly when acetone (boiling point: 56.2 °C and vapor pressure: 240 hPa at 20 °C [38]) was used. This solvent diffused quickly to the external phase and its evaporation led to instability and fast precipitation, as many microparticles were seen adhered to the walls of the container.

#### 3.2.2. Effect of the Composition of Solvent Blend and Its OP Ratio on Zeta Potential and pH

Acid dissociation constants or pK_a_ values usually are used to analyze and compare the deprotonation state of molecules in aqueous systems [39]. The acid dissociation constant measures the strength of an acid in a solution influencing consequently their solubility. The acid will be stronger as the pK_a_ values decrease [40]. However, this definition cannot be applied using organic solvents and their mixtures with water and other polymers. In this regard, there is still a largely uncharted territory about these complex systems [41].

Analyzing the nanoparticles prepared with the polymer PLGA (50:50), PVA at 2% (*w*/*v*) and THF-EtAc, significantly more neutral absolute values of zeta potential (≤2 mV) were observed (*p* < 0.05) compared with the solvent blend ACE-MEK (Table 3, Experimental design 1; Figure 1c). One explanation to this behavior could be given due to the partial protonation of PLGA (pK_a_ = 3.85) produced using 100% (*v*/*v*) of MEK compared with EtAc. It could be hypothesized that more ionized carboxyl groups of the polymer produced a significant lower absolute value of zeta potential. These results are in agreement with those reported previously using PLGA [42], in which the zeta potential of the particles (~1.6 µm) was highly negative at pH 7.4, and almost zero as pH decreased to 3.0. Nonetheless, this significant difference was only observed for PLGA with carboxyl groups ends, contrasting with the results in this study in which PLGA ester ends was utilized. The increasing addition of ACE into the OP produced even more neutral zeta potential values compared with the addition of THF in this study. This effect to obtain more neutral zeta potential values was more evident using PLGA 50:50, P-407 at 1% (*w*/*v*) and ACE-MEK compared with ACE-EtAc (Table 3, Experimental design 11, Figure 7d). This tendency was not observed using the same polymer with a higher concentration of P-407 (5% *w*/*v*), probably due to the thicker layer of the stabilizer around the particle charge (Experimental design 10, Figure 7b).

In the same way, a similar behavior was found when Eudragit^®^ E100 was used in solvent blends consisting of either ACE-EtAc (Table 3, Experimental design 2 and 3; Figure 1, Figure 2 and Figure 3), Et-EtAc (Table 3, Experimental design 3 and 4; Figure 1, Figure 4 and Figure 5), or ACE-MEK (Table 3, Experimental design 6; Figure 6(b3)). Zeta potential decreased as the ratio of the partially water-miscible solvent into the organic phases decreased and simultaneously the water-miscible solvent increased, but they were enough high to provide physical stability (Figure 1, Figure 2, Figure 3, Figure 4, Figure 5 and Figure 6). In this case, the polymer Eudragit^®^ E100, based on dimethylaminoethyl methacrylate and neutral methacrylic acid esters with a pKa of 7.0–7.3 [43], is soluble at pH 5 and swellable/permeable or partially protonated above of this value [44]. Thus, the same hypothesis could be given relating the partial polymer ionization in the systems (solvents, polymers and water) with more acidic properties, especially when the solvent blends of Et-EtAc or ACE-EtAc were used as OPs at low polymer concentrations (200 mg) [45,46,47]. This effect could be observed using either PVA or P-407 as stabilizers but was not so clear using a higher polymer concentration (Table 3, design 6; Figure 6). Further studies need to be done regarding this hypothesis.

The pH of the final aqueous dispersion of NPs (after the solvent evaporation step) was measured showing higher pH values than the pK_a_ = 3.85 of PLGA and more neutral zeta potential values using the solvent blend ACE-MEK or increasing the ratio of the water-miscible solvent into the OP (Table 3, Experimental design 11, Figure 7e). This suggests a higher partial ionization of the polymer molecules in these systems [45,46,47]. As PLGA is an acid, more ionized carboxyl groups of the polymer are produced at a pH above of its pK_a_, in which 50% of the polymeric molecules are ionized.

On the other hand, batches prepared with Eudragit^®^ E 100 and the solvent blend Et-EtAc (Table 3, Experimental design 4) shown higher pH values of the final NPs aqueous dispersions as the partially water-miscible solvent decreased its ratio into the OP (Et:EtAc pH at 0:100 = 6.83, 25:75 = 7.00, 50:50 = 7.52 and 75:25 = 7.46). The pH increasing and the lowering the NPs zeta potential, indicates a higher solubility of Eudragit^®^ E 100 (which behaves as a pH modifier to increase the microenvironment pH as a weak base [48]). Opposite results were observed with a neutral poly-ε-caprolactone using the emulsification-diffusion method [2]. As different molecular arrangements can be obtained by changing the nature of the solvent [49], it could also be hypothesized that at higher ratios of ethanol (dielectric constant: 24 at 25 °C [38]) into the solvent blend Et-EtAc, more polar parts of the polymer could be located near the water-polymer interface, and few alkyl chains could be positioned facing the external phase where the EtAc, of hydrophobic nature (EtAc dielectric constant: 6.27 at 20 °C [50]), could be displaced.

#### 3.2.3. Effect of the Stirring Rate on Particle Size and PdI

The production of particle sizes below 1000 nm with low PdIs could be obtained using a minimum stirring rate of 1500 rpm with a mechanical stirrer (Table 3, experimental design 5, Figure 6(a1,a2)). Stirring rates below of this value and using magnetic stirring produce polydispersions in the nanoscale and the microscale (Table 2). Using a high-performance dispersing machine at 8000 rpm, narrower and more controlled particle size distributions were obtained compared with those produced using mechanical stirring with a 4-bladed dispersing propeller (Table 3, design 1, Figure 1a,b).

#### 3.2.4. Effect of the Polymer Concentration on Particle Size, PdI and Zeta Potential

Comparing the results obtained using higher polymer concentrations, no significant differences or trends on particle sizes were found, except when EtAc was used alone. However, higher PdI indexes and zeta potential values (*p* < 0.05) were found increasing the Eudragit^®^ E100 concentration at 4% (*w*/*v*, Table 3, design 2, Figure 2 and Figure 3). In general, a higher robustness can be achieved by using this modification compared with the standard emulsification–diffusion method, in which particle size increased using a polymer concentration above 2.5% (*w*/*v*) [2]. Nevertheless, a high variation in the data was observed in batches using 4% (*w*/*v*) of the polymer in this study, probably due to the poor solvent diffusion affected by a higher viscosity of the OP. This restricted solvent diffusion could produce an Ostwald ripening phenomenon in the emulsion, but not enough to increase the particle size as it is reported for the emulsification-diffusion [2] and emulsification-evaporation [17] methods.

#### 3.2.5. Effect of the Stabilizers and Their Concentration on Particle Size and PdI

Analyzing the results of particle size by using Eudragit^®^ E 100 (200 mg), PVA and P-407 at 2–5% (*w*/*v*), noticeable differences were observed. The higher particle sizes were found by using higher ratios of the partially water-miscible solvent EtAc into the OPs compared with its lower ratios, but no trends were found. When an OP ratio of 25:75 or 75:25 was used, a tendency to increase the particle size was found as P-407 concentration increased. On the other hand, the use of PVA produced significant higher particles sizes (*p* < 0.05) at higher ratios of the water-miscible solvent into the OP, compared with those using P-407 at all stabilizer concentrations. Additionally, a slightly tendency by using PVA to decrease the particle size was found, by increasing the water-miscible solvent ratio into the OP (Experimental design 4, Table 3, Figure 4a–d and Figure 5a). The same behavior was found for the PdIs, when higher values were obtained as the PVA or P-407 concentration and partially water-miscible solvent into the OP increased (Experimental design 4, Table 3, Figure 4e and Figure 5b).

Increasing the Eudragit^®^ E 100 concentration at 400 mg and using MEK at 100% (*v*/*v*), an evident decrease in the particle size and PdI (*p* < 0.05) was found by using PVA at 2 instead of 1% (*w*/*v*, Table 3, design 8, Figure 6(d1,d2)). Similar results were found using the emulsification-diffusion [1,51,52,53] and a modified emulsion-solvent evaporation process [17], suggesting that more stabilizer molecules can be placed in the o/w interface decreasing the interfacial tension.

A better performance to obtain the smallest sizes either with Eudragit^®^ E100 (~50 nm) or with PLGA (50:50, ~145 nm) was found using P-407 as stabilizer at low concentrations (1%, *w*/*v*), and decreasing the ratio of the partially miscible solvent into the OP, compared with PVA in all the concentration range (1–5% *w*/*v*; Table 3, Experimental design 12, Figure 7f). Nonetheless, similar particles sizes were found with PLGA (50:50) and P-407 ≤ 1% (*w*/*v*; Table 3, Experimental design 9; Figure S2a,b of the Supplementary Material). Contrarily, the highest sizes and PdIs were found using higher concentrations of P-407 and higher ratios of the partially water-miscible solvent into the OP using either Eudragit^®^ E100 or PLGA (50:50; Table 3, Experimental design 10; Figure S2c,d of the Supplementary Material). One explanation for this behavior is the ability of P-407 (critical micelle concentration of 2.8 × 10^−6^ M) to form monomolecular micelles at low concentrations (10^−4^–10^−5^%), while at higher concentrations, multimolecular aggregates are formed with its hydrophilic polyoxyethylene chains facing the aqueous phases and creating a hydrophobic core [54,55]. The results obtained in this study contrast with the performance of stabilizers (PVA > Poloxamers > Polysorbate 80 > SDS = DTAB) observed to decrease in the particle size by emulsification-diffusion method [56], probably due to the low polymer concentration used in this study. Additionally, using P-407 as stabilizer, more physically stable systems with apparently higher yields were obtained compared with those using PVA, in which many particles remained adhered to the container walls at the end of the manufacture process.

The successful production of stable NPs at low stabilizer concentrations provides the advantage of avoiding their post-purification, reducing the toxicity and consequently their time processing.

#### 3.2.6. Effect of the Stabilizers and Their Concentration on Zeta Potential and pH

Stabilizers in this study were non-ionic, providing a steric stabilization effect between the NPs. Significant lower absolute values of zeta potential (*p* < 0.05) were obtained when their concentration increased (Table 3, Experimental design 4, Figure 4f and Figure 5c; Experimental design 8, Figure 6(d3); Experimental design 9, Figure 7a). This behavior has also been reported for NPs of PLGA by emulsification–diffusion [2], and by a modified emulsion-solvent evaporation process [17].

A pattern to decrease the zeta potential was also observed when P-407 at (1–5% *w*/*v*) and Eudragit^®^ E100 at 200 mg were utilized increasing the water-miscible solvent in the OP. Using 400 mg of Eudragit^®^ E 100, the effect was more noticeable. On the other hand, when PLGA (50:50) was used, this effect could even be observed at stabilizer concentrations below 1% (*w*/*v*). This suggests that the stabilizer is adsorbed on the solvent–water interface in the course of the emulsification step to form the droplets, whereas the remaining quantity prevents the particle aggregation [2]. This remaining quantity could produce a dense steric barrier masking the polymer charge and reducing the electrical behavior of the particles [57]. In addition, a higher pH (*p* < 0.05) of the aqueous nanoparticle dispersion prepared with PLGA and PVA was obtained, compared with P-407, indicating a higher partial ionization of the molecules from the PLGA or a partial hydrolysis of PVA acetate group in the lightly acidic medium [58].

#### 3.2.7. Effect of the Addition Order of the OP and AP in the Emulsification Step on the Size and Zeta Potential

Finally, smaller sizes (≤200 nm) and PdI indexes (≤0.2) were found adding the OP into the AP to form an emulsion O/W compared to the inverse order. The phase inversion from an emulsion W/O to change for one O/W could produce a higher instability in the droplets O/W in the emulsification step, probably due to a faster evaporation of the OP by the stirring rate, producing bigger particle sizes (Table 3, design 7, Figure 6(c1–c3)). Zeta potential results are in agreement with the explanation given above.

Nanoparticles of 50 ≤ 1000 nm could be successfully produced by a rapid emulsion-diffusion method using feasible operating conditions and equipment. A useful linear correlation between the particle size and the solubility parameter was found. Nonetheless, further studies must be performed to analyze the correlation of all solubility parameters from each formulation component versus the particle size to confirm the results obtained in this investigation. In addition, studies to entrap drugs in these NPs and to confirm their particle size, as well as to know their shape, texture, and structure need to be achieved to confirm its feasibility.

## 4. Conclusions

A rapid emulsion-diffusion method to broadly prepare polymeric nanoparticles by using safe materials and solvent blends (miscible/partially miscible in water, 25–100%) as OPs is presented. No previous mutual organic/aqueous phase saturation and no water addition after the emulsification step were required. Emulsification of the OP dropwise addition into the AP or vice versa using low-high shear stirring produced unimodal nanoparticle distributions. An important linear correlation was found to predict the particle size from the OP solubility parameters. The decrease in the partially water-miscible solvent ratio or its polarity when they were used at 100% (*v*/*v*), or lowering the P-407 concentrations; or the increase in the OP polarity or concentrations of PVA produced smaller sizes and PdIs using either Eudragit^®^ E 100 or PLGA (50:50). The smallest (~50 nm) and highest sizes (≥600 nm) and PdIs were obtained using P-407 compared with the use of PVA. Absolute values of zeta potential decreased when the water-miscible solvent in the OP or stabilizer concentration increased. An understanding of the influence on the formulation and processing parameters is given to feasibly produce NPs using simple equipment and safe materials. More studies should be conducted entrapping drugs.

## Figures and Tables

**Table 1:** Steps of the emulsion-diffusion method modifications.

Solvent Type of OP	Saturation	Emulsification			Dilution (Water)	EVP	NP Type/Ref.
		Phases Addition	Mixing	S			
**Standard emulsification-diffusion method**							
Partially water-miscible solvents	+	OP → AP	High-shear stirring	+	+	+/F	NSP [1]
**Modifications of the emulsification-diffusion method**							
Partially water-miscible solvents	+	OP → AP	Low/high shear stirring	+	−	+	NSP [8]
Partially water-miscible solvents	+ (47 °C)	OP → AP	High-shear stirring (47 ± 2 °C)	+	+	+	SLN [9]
Miscible, non-miscible and partially miscible solvents in water (individual or combined)	−	OP → AP (AP: glucose–ACE)	Pre-stirring (1000 rpm/3 h) High-shear stirring (15,000 rpm/5 min)	+	+	+	NSP [11]
Partially water-miscible solvent	−	OP → AP	Pre-stirring (3 h) High-shear stirring (15,000 rpm/5 min)	+	+ (water bath 40 °C)	+	[10] NSP
Partially water-miscible solvent	−	OP → AP	Pre-stirring (1000 rpm/20 min) → High-shear stirring (10,000–15,000 rpm) or Sonication (60 % amplitude/1 min)	+	+	+	NSP [12]
Partially water-miscible solvent	+	OP → AP	Ultrasound (2 min), ice bath	+	+	+ (40 °C)	NC [13]
Two organic phases: 1. Partially water-miscible gas + drug/ 2. Water-miscible solvent + polymer	−	Two steps: 1 = OP_1_ → OP_2_ 2 = OP_2_ → AP 2 mL/min	Magnetic stirring	+	+	+ (3 h)	NC [14]
Partially water-miscible solvent	−	AP → OP	Magnetic stirring	˗	−	C	MCP [15]
**Modifications of the emulsification-solvent evaporation method**							
Partially miscible or non-miscible solvents in water	−	OP → AP	Ultrasound (55 W/1 min)	+	−	+	NSP [16]
Blends of solvents miscible and non-miscible in water	−	OP → AP	Ultrasound (50 W/30 s, in an ice bath)	+	−	+	NSP [17]
Blends of solvents miscible and non-miscible in water	−	Two steps: 1: OP → AP_1_ 2: AP_1_ → AP_2_	Step 1: High stirring (17,500 rpm/5 min), Step 2: Magnetic stirring (40 °C/40 min).	+	−	+ (40 °C)	NSP, NC and NE [18]
**Modification of the emulsification-diffusion method proposed in this study**							
Blends of solvents miscible and partially miscible in water	−	OP → AP or AP → OP (2 mL/min)	Low/medium shear stirring (>1500–8000 rpm)	+	−	+ (30 °C)	NSP

+: Yes; −: Not; OP: Organic phase; AP: Aqueous phase; EVP: Evaporation; S: Use of Stabilizers; NSP: Nanospheres; SLN: Solid lipid nanoparticles; NC: Nanocapsules; MCP: Microcapsules and NE: Nanoemulsions; ACE: Acetone; F: Filtration; C: Centrifugation.

**Table 2:** Nanoparticle optimization utilizing polymer, organic phase, and manufacturing parameters.

Batch	Stabilizer % (*w*/*v*)	PLGA Type, % (*w*/*v*)	AP (mL)	OP % (*v*/*v*) mL	Stirring	Ratio OP:AP
***a***	PVA (2.0)	(85:15), 1	0	Et-PEG (90:10) 20	*	(1:0)
			0	Et-PEG (80:20) 20	*	
		(85:15), 1.4	0	Et-PEG-THF (57.14:14.29:28.57) 28	*	(1.4:0)
		(85:15), 1.8	0	Et-PEG-THF (44.44:11.11:44.44) 36	*	(1.8:0)
		(85:15), 1.4	40	Et-PEG-THF (36.36:9.509:54.54) 44	Magnetic	(1.05:0.95)
***b***	PVA (2.0)	(85:15), 2.2	40	Et-MC (50:50) 20	Magnetic	(1:2)
***c***	PVA (0.4545)	(50:50), 1	44	EtAc-Et (71.43:28.57) 20	Magnetic	(0.77:1.22)
***d***	PVA (2.0)	(50:50), 1	40	THF-PEG (80:20) 20	Magnetic	(1:2)
***e***	PVA (2.0)	(50:50), 1	40	THF (100) 20	Magnetic	(1:2)
***f***	PVA (2.0)	(50:50), 1	40	THF-EtAc-PEG (70:25:5) 20	Magnetic	(1:2)
***g***	PVA (2.0)	(50:50), 1	40	THF-EtAc (75:25) 20	Magnetic 10 min + UT 8000 rpm, 10 min	(1:2)
***h***	PVA (2.0)	(50:50), 0.5	20	THF (100) 20	Magnetic	(1:1)
***i***	PVA (2.0)	(50:50), 0.5	40	THF (100) 20	Magnetic	(1:2)
***j***	PVA (2.0)	(50:50), 1	40	THF-EtAc (50:50) 20	UT, 8000 rpm *	(1:2)

PLGA: Poly (DL-lactide-co-glycolide) 85:15 and 50:50; PEG: polyethylene glicol 400; Et: MC: methylene chloride; THF: tetrahydrofuran; EtAc: ethyl acetate; UT: Ultra-Turrax^®^ homogenizer. * Incomplete solubilization, emulsification step was not followed, (*n* = 3).

**Table 3:** Experimental designs performed using the rapid emulsification-diffusion method.

Design	Polymer (mg)	OP Ratio (*v*/*v*)	Stabilizer (% *w*/*v*)	Stirring Rate (rpm)	Factors	Levels					
1	PLGA (50:50) (200 mg)	-	PVA (2%)	8000 ^a^	OP ratio	75:25	50:50		25:75		0:100
					Type of OP	THF-EtAc			ACE-MEK		
2	Eudragit^®^ E 100 (−-)	ACE-EtAc	PVA (2%)	2000 ^b^	[Polymer]	200 mg		400 mg		800 mg	
					OP ratio	75:25	50:50		25:75		0:100
3	Eudragit^®^ E 100 (200 mg)	-	PVA (2%)	2000 ^b^	water miscible solvent in the OP	ACE-EtAc			Et-EtAC		
					OP ratio	75:25	50:50		25:75		0:100
4	Eudragit^®^ E 100 (200 mg)	Et-EtAc	-	2000 ^b^	[Stabilizer]	1% (*w*/*v*)		2% (*w*/*v*)		5% (*w*/*v*)	
					Type of stabilizer	PVA			P-407		
					OP ratio	75:25	50:50		25:75		0:100
5	Eudragit^®^ E 100 (200 mg)	MEK (100)	PVA (2%)	-	Stirring rate	500	1500		2000		3000
						(rpm)					
6	Eudragit^®^ E 100 (400 mg)	ACE-MEK	PVA (2%)	1500 ^b^	OP ratio	75:25	50:50		25:75		0:100
7	PLGA (50:50) (200 mg)	ACE-MEK (50:50)	PVA (2%)	8000 ^a^	AP:OP order of addition	OP added to the AP AP added to the OP					
8	Eudragit^®^ E 100 (400 mg)	MEK (100)	-	1500 ^b^	[PVA]	1% (*w*/*v*)			2% (*w*/*v*)		
9	PLGA (50:50) (200 mg)	ACE-MEK (75:25)	-	2000	[P-407]	0.25% (*w*/*v*)		0.5% (*w*/*v*)		1% (*w*/*v*)	
10	PLGA (50:50) (200 mg)	ACE-EtAc	P-407 (5%)	2000	OP ratio	25:75			50:50		
11	PLGA (50:50) (200 mg)	-	P-407 (1%)	2000	OP ratio	25:75			75:25		
					Type of OP	ACE-EtAc			ACE-MEK		
12	PLGA (50:50) (200 mg)	ACE-MEK (75:25)	(1%)	2000	Type of stabilizer	PVA			P-407		

PLGA: Poly (DL-lactide-*co*-glycolide) 50:50; OP ratio: ratios of the water miscible solvent and a partially water-miscible solvent; PVA: polyvinyl alcohol; ACE: acetone; MEK: methyl ethyl ketone; P-407: Poloxamer 407. All the systems were prepared with OP: 20 mL; AP: 40 mL; (*n* = 3). ^a^ Systems emulsified with Ultra-Turrax^®^. ^b^ Systems emulsified with a mechanical stirrer and a 4-bladed propeller stirrer.

**Table 4:** Hansen solubility parameters of pure solvents and solvent blends used to prepare nanoparticles.

Pure Solvents and Solvent Blends	Hansen Solubility Parameter (MPa^1/2^)			
	*δ_D_*	*δ_P_*	*δ_H_*	*δ_HaSP_*
ACE 100%	15.5	10.4	7	19.9
EtAc 100%	15.2	5.3	9.2	18.5
MEK 100%	15.9	9	5.1	19.0
THF 100%	16.8	5.7	8	19.5
Et 100%	15.8	8.8	19.5	26.6
ACE-MEK (0:100)	15.9	9.0	5.1	19.0
ACE-MEK (25:75)	15.8	9.4	5.6	19.2
ACE-MEK (50:50)	15.7	9.7	6.1	19.4
ACE-MEK (75:25)	15.6	10.1	6.5	19.7
THF-EtAc (0:100)	15.2	5.3	9.2	18.6
THF-EtAc (25:75)	15.6	5.4	8.9	18.8
THF-EtAc (50:50)	16.0	5.5	8.6	19.0
THF-EtAc (75:25)	16.4	5.6	8.3	19.2
ACE-EtAc (0:100)	15.2	5.3	9.2	18.6
ACE-EtAc (25:75)	15.3	6.6	8.7	18.7
ACE-EtAc (50:50)	15.4	7.9	8.1	19.0
ACE-EtAc (75:25)	15.4	9.1	7.6	19.4
Et-EtAc (0:100)	15.2	5.3	9.2	18.6
Et-EtAc (25:75)	15.4	6.2	11.8	20.3
Et-EtAc (50:50)	15.5	7.1	14.4	22.3
Et-EtAc (75:25)	15.7	7.9	16.9	24.4

Solubility parameters of the pure solvents were obtained from the literature [31] and the solubility parameters of the solvent blends were calculated as described in the method section.

**Table 5:** Linear correlation data from Hansen solubility parameters of OPs versus nanoparticle size.

Solvent Blend or OP	[Polymer]	[Stabilizer] % (*w*/*v*)	Correlation Equation for Particle Size ^a^	r^2^
ACE-MEK (0:100 to 75:25)	PLGA (50:50), 200 mg	PVA 2%	*y* = −427.93*x* + 8556.4	0.8432
THF-EtAc (0:100 to 75:25)			*y* = −157.57*x* + 3201.8	0.7699
ACE-EtAc (0:100 to 75:25)	Eudragit^®^ E100, 200 mg		*y* = −124.29*x* + 2590.2	0.8197
	Eudragit^®^ E100, 400 mg		*y* = −130.23*x* + 2723.1	0.2000
	Eudragit^®^ E100, 800 mg		*y* = 8135.6*x* − 144,556	0.0227
ACE-MEK (0:100 to 75:25)	Eudragit^®^ E100, 400 mg		*y* = −739.64*x* + 14,788	0.1503
Et-EtAc (0:100 to 75:25)	Eudragit^®^ E100, 200 mg	PVA 1%	*y* = −2.7458*x* + 238.35	0.0281
		PVA 2%	*y* = −20.285*x* + 642.2	0.9037
		PVA 5%	*y* = −7.8519*x* + 338.56	0.1376
		P-407 1%	*y* = −35.846*x* + 917.08	0.9016
		P-407 2%	*y* = −26.586*x* + 726.96	0.3481
		P-407 5%	*y* = −64.851*x* + 1686.3	0.2465

All systems were prepared with an OP: 20 mL and an AP: 40 mL; (*n* = 3). **^a^** The general linear equation is *y = mx + b*, where *y* is the particle size for a solubility parameter given (*x*).

## Supplementary Materials

The following are available online at https://www.mdpi.com/article/10.3390/nano12020229/s1, Figure S1: (**a**) Batches showing the particle size versus PdI and (**b**) particle size versus the zeta potential in the optimization of the biodegradable NPs (PLGA 85:15 and 50:50 according to Table 2) of using different variables reported in Table 2. Bars error = SD, *n* = 3; Figure S2: Influence of P-407 at three low concentrations using ACE-MEK (75:25) on (**a**) the particle size and (**b**) PdI (Experimental design 9). Effect of ACE-EtAc at two ratios on the (**c**) particle size and (**d**) PdI, using P-407 5% (*w*/*v*; Experimental design 10). Influence of two solvent blends, as OP, at two ratios on the (**e**) PdI, by using P-407 1% as stabilizer (Experimental design 11). Analysis of the effect of P-407 and PVA at 1% on the (**f**) PdI, using the solvent blend ACE-MEK (75:25), Experimental design 12. All batches were prepared with 200 mg of PLGA (50:50), 2000 rpm, and a ratio of OP:AP (1:2), *n* = 3. All the means bars correspond to the Bonferroni interval at the 95.0% confidence level. Table S1. Particle size, Zeta potential and PdI of all batches prepared at different formulation and operating conditions.
