# Supplementary material for: Effects of Process and Formulation Parameters on Submicron Polymeric Particles Produced by a Rapid Emulsion-Diffusion Method
Source: Nanomaterials (Basel). 2022 Jan 11;12(2):229. doi: 10.3390/nano12020229 (PMC8780531; doi:10.3390/nano12020229)
Supplement: Supplementary file 1 [file nanomaterials-12-00229-s001.zip › nanomaterials-1495098-supplementary.pdf]

Article

Effects of Process and Formulation Parameters on Submicron Polymeric Particles Produced by a Rapid Emulsion-Diffusion Method

Clara Luisa Domínguez-Delgado *, Zubia Akhtar, Godfrey Awuah-Mensah, Braden Siyi Wu and Hugh David Charles Smyth *

Division of Molecular Pharmaceutics and Drug Delivery, College of Pharmacy, The University of Texas at Austin, 2409 University Avenue, Austin, TX 78712, USA; zubiaakhtar@utexas.edu (Z.A.); gamensah@utexas.edu (G.A.-M.); braden.s.wu@gmail.com (B.S.W.)

* Correspondence: claraldominguezd@gmail.com (C.L.D.-D.); hugh.smyth@austin.utexas.edu (H.D.C.S.); Tel.: +52-553-489-9461 (C.L.D.-D.); +1-512-471-3383 (H.D.C.S.)

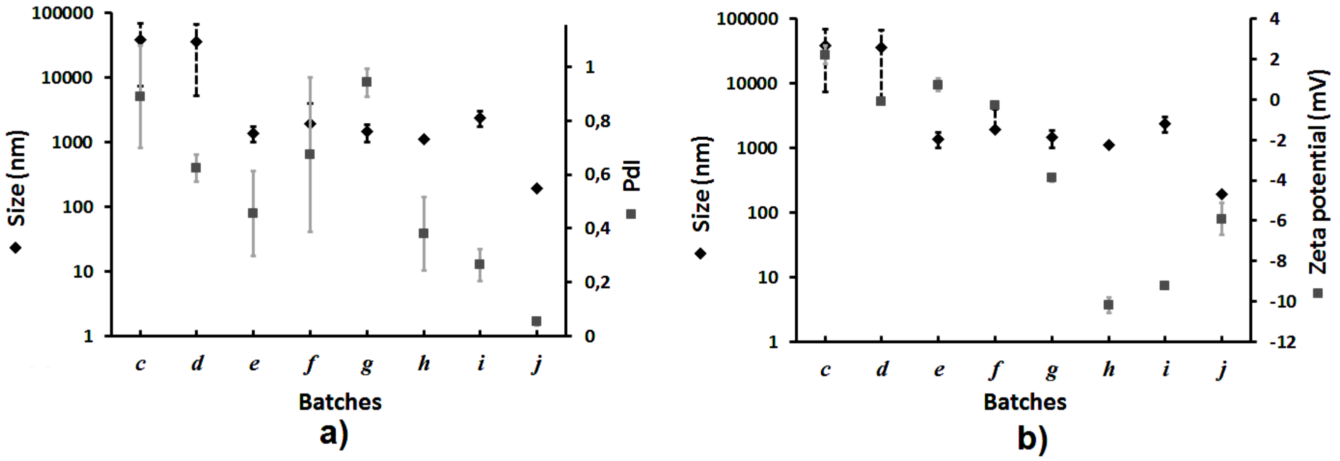

Figure S1. **a)** Batches showing the particle size versus PdI and **b)** particle size versus the zeta potential in the optimization of the biodegradable NPs (PLGA 85:15 and 50:50) of using different variables reported in Table 2. Bars error =SD, n=3.

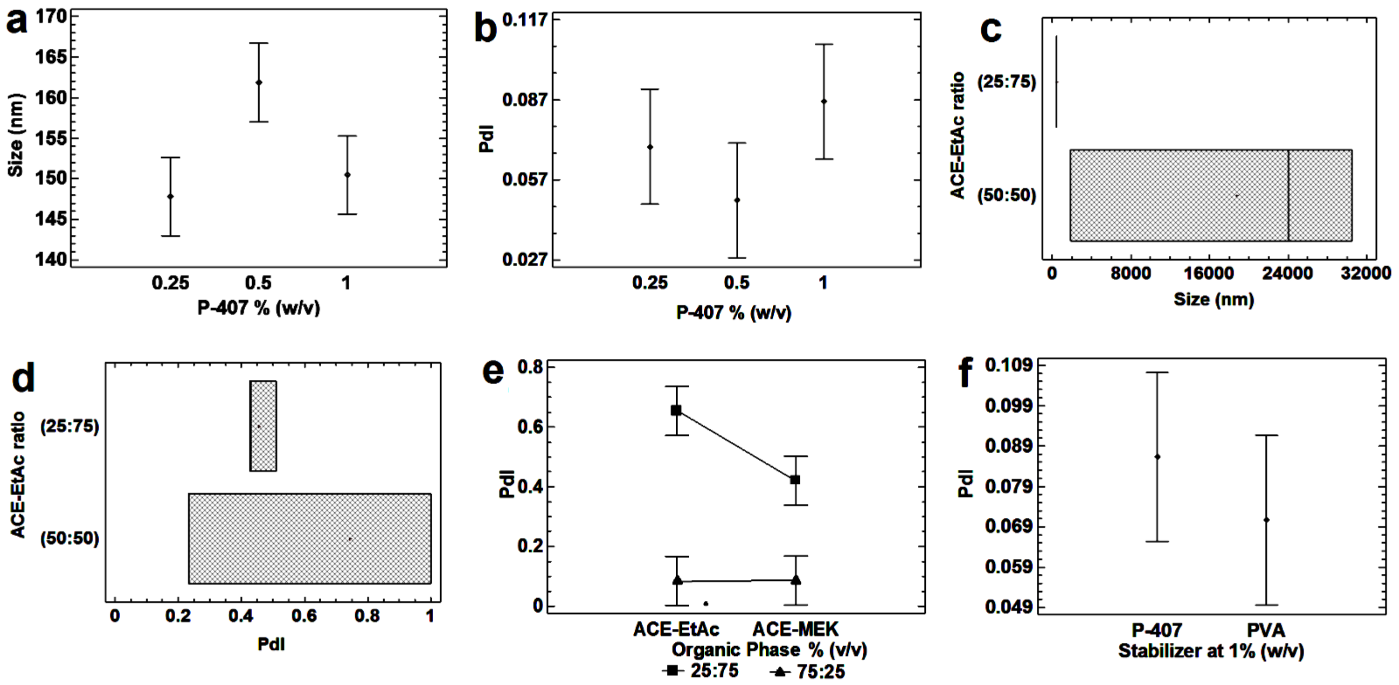

Figure S2. Influence of P-407 at three low concentrations using ACE-MEK (75:25) on **a)** the particle size and **b)** PdI (Experimental design 9). Effect of ACE-EtAc at two ratios on the **c)** particle size and **d)** PdI, using P-407 5% (w/v; Experimental design 10). Influence of two solvent blends, as OP, at two ratios on the **e)** PdI, by using P-407 1% as stabilizer (Experimental design 11). Analysis of the effect of P-407 and PVA at 1% on the **f)** PdI, using the solvent blend ACE-MEK (75:25; Experimental design 12). All batches were prepared with 200mg of PLGA (50:50), 2000 rpm, and a ratio of OP:AP (1:2), n=3. All the means bars correspond to the Bonferroni interval at the 95.0% confidence level.

Table S1. Particle size, Zeta potential and PdI of all batches prepared at different formulation and operating conditions.

Polymer (mg)	Organic Phase ratio (v/v)	Stabilizer (% w/v)	Stirring rate (rev/min)	Stirrer	OP:AP ratio	Mean size (nm) ± SD			Mean PdI ± SD			Mean Z Potential (mV) ± SD			Mean pH ± SD
PLGA (50:50) (200 mg)	THF-EtAc (50:50)	PVA 2%	8000	Ultraturrax	(1:2)	189,73	±	4,10	0,054	±	0,01	-5,92	±	0,79	_
PLGA (50:50) (200 mg)	THF-EtAc (75:25)	PVA 2%	8000	Ultraturrax	(1:2)	196,60	±	0,70	0,053	±	0,01	-5,23	±	0,13	_
PLGA (50:50) (200 mg)	THF-EtAc (25:75)	PVA 2%	8000	Ultraturrax	(1:2)	223,67	±	6,71	0,058	±	0,01	-5,55	±	0,19	_
PLGA (50:50) (200 mg)	EtAc (100)	PVA 2%	8000	Ultraturrax	(1:2)	308,53	±	10,36	0,113	±	0,02	-6,75	±	0,43	_
PLGA (50:50) (200 mg)	ACE-MEK (50:50)	PVA 2%	8000	Ultraturrax	(1:2)	181,17	±	2,86	0,083	±	0,01	-0,71	±	0,05	_
PLGA (50:50) (200 mg)	ACE-MEK (75:25)	PVA 2%	8000	Ultraturrax	(1:2)	163,67	±	3,70	0,068	±	0,01	-3,88	±	0,40	_

PLGA (50:50) (200 mg)	ACE-MEK (25:75)	PVA 2%	8000	Ultraturrax	(1:2)	400,70	±	15,55	0,259	±	0,02	-0,40	±	0,19	_
PLGA (50:50) (200 mg)	MEK (100)	PVA 2%	8000	Ultraturrax	(1:2)	409,90	±	24,97	0,583	±	0,06	2,08	±	0,47	_
Eudragit E (200 mg)	ACE-EtAc (50:50)	PVA 2%	2000	Mechanical S.	(1:2)	254,20	±	10,93	0,356	±	0,06	13,23	±	1,79	_
Eudragit E (200 mg)	ACE-EtAc (75:25)	PVA 2%	2000	Mechanical S.	(1:2)	155,33	±	2,85	0,092	±	0,01	20,27	±	2,98	_
Eudragit E (200 mg)	ACE-EtAc (25:75)	PVA 2%	2000	Mechanical S.	(1:2)	258,70	±	11,11	0,295	±	0,02	42,60	±	1,47	_
Eudragit E (200 mg)	EtAc (100)	PVA 2%	2000	Mechanical S.	(1:2)	278,90	±	10,74	0,238	±	0,01	38,80	±	0,85	_
Eudragit E (400 mg)	ACE-EtAc (50:50)	PVA 2%	2000	Mechanical S.	(1:2)	343,67	±	164,57	0,431	±	0,04	39,70	±	1,51	_

Eudragit E (400 mg)	ACE-EtAc (75:25)	PVA 2%	2000	Mechanical S.	(1:2)	147,23	±	0,74	0,156	±	0,03	38,33	±	1,00	_
Eudragit E (400 mg)	ACE-EtAc (25:75)	PVA 2%	2000	Mechanical S.	(1:2)	221,70	±	6,82	0,362	±	0,02	39,83	±	1,39	_
Eudragit E (400 mg)	EtAc (100)	PVA 2%	2000	Mechanical S.	(1:2)	315,70	±	13,17	0,270	±	0,00	42,50	±	0,66	_
Eudragit E (800 mg)	ACE-EtAc (50:50)	PVA 2%	2000	Mechanical S.	(1:2)	16578,77	±	16272,28	0,849	±	0,23	66,83	±	4,06	_
Eudragit E (800 mg)	ACE-EtAc (75:25)	PVA 2%	2000	Mechanical S.	(1:2)	156,57	±	4,75	0,153	±	0,01	33,97	±	0,25	_
Eudragit E (800 mg)	ACE-EtAc (25:75)	PVA 2%	2000	Mechanical S.	(1:2)	353,33	±	204,67	0,420	±	0,10	51,53	±	3,10	_

Eudragit E (800 mg)	EtAc (100)	PVA 2%	2000	Mechanical S.	(1:2)	346,33	±	11,86	0,298	±	0,02	41,17	±	0,06	_
Eudragit E (400 mg)	ACE-MEK (50:50)	PVA 2%	1500	Mechanical S.	(1:2)	196,97	±	3,31	0,227	±	0,02	37,77	±	0,64	_
Eudragit E (400 mg)	ACE-MEK (75:25)	PVA 2%	1500	Mechanical S.	(1:2)	177,63	±	4,58	0,153	±	0,00	47,37	±	1,53	_
Eudragit E (400 mg)	ACE-MEK (25:75)	PVA 2%	1500	Mechanical S.	(1:2)	1325,33	±	58,76	0,640	±	0,06	50,43	±	3,43	_
Eudragit E (400 mg)	MEK (100)	PVA 2%	1500	Mechanical S.	(1:2)	293,50	±	8,11	0,269	±	0,00	22,23	±	3,18	_
Eudragit E (200 mg)	Et-EtAc (50:50)	PVA 2%	2000	Mechanical S.	(1:2)	172,57	±	2,83	0,122	±	0,03	15,07	±	1,59	_
Eudragit E (200 mg)	Et-EtAc (75:25)	PVA 2%	2000	Mechanical S.	(1:2)	161,70	±	4,30	0,078	±	0,02	30,50	±	1,93	_

Eudragit E (200 mg)	Et-EtAc (25:75)	PVA 2%	2000	Mechanical S.	(1:2)	222,27	±	6,07	0,192	±	0,01	15,07	±	0,67	_
Eudragit E (200 mg)	EtAc (100)	PVA 2%	2000	Mechanical S.	(1:2)	278,90	±	10,74	0,238	±	0,01	38,80	±	0,85	_
Eudragit E (200 mg)	Et-EtAc (50:50)	PVA 1%	2000	Mechanical S.	(1:2)	198,03	±	13,05	0,283	±	0,02	21,13	±	0,87	_
Eudragit E (200 mg)	Et-EtAc (75:25)	PVA 1%	2000	Mechanical S.	(1:2)	175,80	±	5,52	0,225	±	0,01	45,70	±	1,04	_
Eudragit E (200 mg)	Et-EtAc (25:75)	PVA 1%	2000	Mechanical S.	(1:2)	124,93	±	4,57	0,210	±	0,03	36,20	±	5,78	_
Eudragit E (200 mg)	EtAc (100)	PVA 1%	2000	Mechanical S.	(1:2)	220,00	±	6,24	0,315	±	0,02	43,63	±	1,68	_

Eudragit E (200 mg)	Et-EtAc (50:50)	PVA 5%	2000	Mechanical S.	(1:2)	221,63	±	11,31	0,568	±	0,07	22,57	±	0,32	_
Eudragit E (200 mg)	Et-EtAc (75:25)	PVA 5%	2000	Mechanical S.	(1:2)	127,33	±	10,70	0,467	±	0,04	23,53	±	0,67	_
Eudragit E (200 mg)	Et-EtAc (25:75)	PVA 5%	2000	Mechanical S.	(1:2)	122,80	±	1,08	0,259	±	0,01	23,87	±	2,61	_
Eudragit E (200 mg)	EtAc (100)	PVA 5%	2000	Mechanical S.	(1:2)	211,50	±	3,92	0,205	±	0,01	29,20	±	0,26	_
Eudragit E (200 mg)	Et-EtAc (50:50)	P-407 1%	2000	Mechanical S.	(1:2)	118,23	±	7,77	0,338	±	0,07	42,40	±	3,77	_
Eudragit E (200 mg)	Et-EtAc (75:25)	P-407 1%	2000	Mechanical S.	(1:2)	49,43	±	0,76	0,181	±	0,02	26,13	±	3,93	_
Eudragit E (200 mg)	Et-EtAc (25:75)	P-407 1%	2000	Mechanical S.	(1:2)	170,47	±	5,98	0,406	±	0,06	44,37	±	0,58	_

Eudragit E (200 mg)	EtAc (100)	P-407 1%	2000	Mechanical S.	(1:2)	267,07	±	55,74	0,474	±	0,10	26,70	±	4,61	_
Eudragit E (200 mg)	Et-EtAc (50:50)	P-407 2%	2000	Mechanical S.	(1:2)	112,47	±	2,85	0,264	±	0,01	34,83	±	1,31	_
Eudragit E (200 mg)	Et-EtAc (75:25)	P-407 2%	2000	Mechanical S.	(1:2)	53,47	±	0,20	0,153	±	0,01	22,67	±	0,67	_
Eudragit E (200 mg)	Et-EtAc (25:75)	P-407 2%	2000	Mechanical S.	(1:2)	316,60	±	34,91	0,545	±	0,02	37,80	±	2,00	_
Eudragit E (200 mg)	EtAc (100)	P-407 2%	2000	Mechanical S.	(1:2)	153,50	±	3,21	0,440	±	0,05	37,20	±	3,56	_
Eudragit E (200 mg)	Et-EtAc (50:50)	P-407 5%	2000	Mechanical S.	(1:2)	128,60	±	3,38	0,297	±	0,03	32,57	±	1,04	_
Eudragit E (200 mg)	Et-EtAc (75:25)	P-407 5%	2000	Mechanical S.	(1:2)	65,68	±	1,08	0,177	±	0,00	26,57	±	6,64	_

Eudragit E (200 mg)	Et-EtAc (25:75)	P-407 5%	2000	Mechanical S.	(1:2)	735,77	±	281,26	0,805	±	0,03	34,20	±	2,08	_
Eudragit E (200 mg)	EtAc (100)	P-407 5%	2000	Mechanical S.	(1:2)	273,67	±	13,64	0,485	±	0,03	31,50	±	0,30	_
PLGA (50:50) (200 mg)	ACE-MEK (50:50)	PVA 2%	8000	Ultraturrax	(1:2)	181,17	±	2,86	0,083	±	0,01	-0,71	±	0,05	_
PLGA (50:50) (200 mg)	ACE-MEK (50:50)	PVA 2%	8000	Ultraturrax	(1:2 inverted)	327,73	±	2,94	0,460	±	0,08	-3,75	±	0,15	_
Eudragit E (200 mg)	MEK (100)	PVA 2%	500	Mechanical S.	(1:2)	7304,67	±	837,09	1,000	±	0,00	33,77	±	7,39	_
Eudragit E (200 mg)	MEK (100)	PVA 2%	1500	Mechanical S.	(1:2)	706,37	±	310,40	0,544	±	0,18	42,70	±	3,25	_

Eudragit E (200 mg)	MEK (100)	PVA 2%	2000	Mechanical S.	(1:2)	574,80	±	172,15	0,596	±	0,17	21,73	±	3,01	_
Eudragit E (200 mg)	MEK (100)	PVA 2%	3000	Mechanical S.	(1:2)	853,27	±	861,67	0,626	±	0,32	34,83	±	6,08	_
Eudragit E (400 mg)	EtAc (100)	PVA 2%	2000	Mechanical S.	(1:2)	315,70	±	13,17	0,270	±	0,00	42,50	±	0,66	_
Eudragit E (200 mg)	EtAc (100)	PVA 2%	2000	Mechanical S.	(1:2)	278,90	±	10,74	0,238	±	0,01	38,80	±	0,85	_
PLGA (85:15) (200)	EtAc (100)	PVA 2%	2000	Mechanical S.	(1:2)	355,03	±	4,75	0,261	±	0,02	1,70	±	0,75	_
PLGA (85:15) (200 mg)	THF-EtAc (50:50)	PVA 2%	8000	Ultraturrax	(1:4)	329,07	±	11,55	0,247	±	0,01	-3,48	±	0,54	_
PLGA (85:15) (200 mg)	THF-EtAc (75:25)	PVA 2%	11000	Ultraturrax	(1:6)	490,40	±	3,82	0,915	±	0,07	-3,97	±	0,19	_

Eudragit E (400 mg)	MEK (100)	PVA 1%	1500	Mechanical S.	(1:2)	719,10	±	63,75	0,641	±	0,19	47,93	±	0,84		_	
Eudragit E (200 mg)	THF-EtAc (50:50)	PVA 2%	2000	Mechanical S.	(1:2)	256,60	±	6,51	0,201	±	0,01	26,60	±	0,61		_	
PLGA (50:50), 200 mg	Ac-EtAc (75:25)	P-407 1%	2000	Mechanical S.	(1:2)	174,00	±	5,03	0,083	±	0,03	-29,83	±	1,45	4,38	±	0,07
PLGA (50:50), 200 mg	Ac-EtAc (25:75)	P-407 1%	2000	Mechanical S.	(1:2)	590,37	±	39,13	0,655	±	0,10	-37,67	±	0,76	3,67	±	0,05
PLGA (50:50), 200 mg	Ac-EtAc (50:50)	P-407 5%	2000	Mechanical S.	(1:2)	18787,00	±	15014 ,31	0,745	±	0,44	-35,17	±	0,60	3,98	±	0,02
PLGA (50:50), 200 mg	Ac-EtAc (25:75)	P-407 5%	2000	Mechanical S.	(1:2)	465,27	±	6,79	0,456	±	0,05	-32,40	±	1,11	3,94	±	0,05
PLGA (50:50), 200 mg	Ac-MEK (25:75)	P-407 1%	2000	Mechanical S.	(1:2)	400,53	±	14,44	0,420	±	0,04	-25,80	±	2,25	3,97	±	0,06

PLGA (50:50), 200 mg	Ac-MEK (75:25)	P-407 1%	2000	Mechanical S.	(1:2)	150,47	±	5,41	0,086	±	0,01	-23,43	±	0,23	4,11	±	0,10
PLGA (50:50), 200 mg	Ac-MEK (50:50)	P-407 1%	2000	Mechanical S.	(1:2)	175,27	±	0,96	0,069	±	0,02	-30,40	±	1,39	3,86	±	0,02
PLGA (50:50), 200 mg	Ac-MEK (75:25)	P-407 0.5%	2000	Mechanical S.	(1:2)	161,87	±	2,65	0,049	±	0,02	-30,23	±	0,95		_	
PLGA (50:50), 200 mg	Ac-MEK (75:25)	PVA 1%	2000	Mechanical S.	(1:2)	163,27	±	2,86	0,071	±	0,02	-16,13	±	0,78	4,49	±	0,01
PLGA (50:50), 200 mg	Ac-MEK (75:25)	P-407 0.25%	2000	Mechanical S.	(1:2)	147,80	±	1,67	0,069	±	0,01	-36,23	±	0,81	3,73	±	0,01
